# High diversity of airborne fungi in the hospital environment as revealed by meta-sequencing-based microbiome analysis

**DOI:** 10.1038/srep39606

**Published:** 2017-01-03

**Authors:** Xunliang Tong, Hongtao Xu, Lihui Zou, Meng Cai, Xuefeng Xu, Zuotao Zhao, Fei Xiao, Yanming Li

**Affiliations:** 1 Department of Geriatrics, Beijing Hospital, National Center of Gerontology, Beijing, The People’s Republic of China; 2 Department of Laboratory Medicine, Beijing Hospital, Beijing, The People’s Republic of China; 3 Key Laboratory of Geriatrics, Beijing Institute of Geriatrics, Beijing Hospital, Beijing, The People’s Republic of China; 4 Department of Hospital Infection Control and Management, Beijing Hospital, Beijing, The People’s Republic of China; 5 National Clinical Research Centre for Respiratory Medicine, Beijing Hospital, Beijing, The People’s Republic of China; 6 Department of Dermatology, First Hospital, Peking University, Beijing, The People’s Republic of China; 7 Department of Respiratory and Critical Care Medicine, Beijing Hospital, National Center of Respiratory, Beijing, The People’s Republic of China

## Abstract

Invasive fungal infections acquired in the hospital have progressively emerged as an important cause of life-threatening infection. In particular, airborne fungi in hospitals are considered critical pathogens of hospital-associated infections. To identify the causative airborne microorganisms, high-volume air samplers were utilized for collection, and species identification was performed using a culture-based method and DNA sequencing analysis with the Illumina MiSeq and HiSeq 2000 sequencing systems. Few bacteria were grown after cultivation in blood agar. However, using microbiome sequencing, the relative abundance of fungi, Archaea species, bacteria and viruses was determined. The distribution characteristics of fungi were investigated using heat map analysis of four departments, including the Respiratory Intensive Care Unit, Intensive Care Unit, Emergency Room and Outpatient Department. The prevalence of *Aspergillus* among fungi was the highest at the species level, approximately 17% to 61%, and the prevalence of *Aspergillus fumigatus* among *Aspergillus* species was from 34% to 50% in the four departments. Draft genomes of microorganisms isolated from the hospital environment were obtained by sequence analysis, indicating that investigation into the diversity of airborne fungi may provide reliable results for hospital infection control and surveillance.

The increasing number of healthcare-associated infections has received significant attention in recent decades, especially opportunistic fungal infections^1^,^2^. The number of invasive fungal infections acquired at the hospital is increased, and the lives of these patients are threatened^2^. The routes of infection transmission include airborne transmission, contact transmission, common vehicles, medical devices and instrumentation^3^. Airborne infection is considered a major route of transmission in hospitals, and the prevention of airborne pathogens is critically important to control hospital infections^3^,^4^. Effective environmental monitoring could be helpful in reducing the infection rates at hospitals, although the lack of developed methodologies for monitoring changes in airborne pathogens restricts the management and control of hospital infections^5^.

Recently, 16 S rRNA sequencing techniques have shown great advantages to survey the diversity of bacterial genera in indoor air^6^,^7^ compared to the traditional culture-based method. Besides, the metagenomic sequencing could allow the alignment of millions of reads to reconstruct individual genomes^8^ and metabolic signatures^9^, which adds much more valuable information to the study of indoor airborne microbiomes and the effect on the inhabitants. Metagenomic profiles of airborne microbiome have been analyzed in a large built environment^10^, on a city-wide scale^11^ and shown to transfer with inhabitants to new homes^12^, which provide comprehensive information about the diversity and abundance of microbiome.

Traditional methods of hospital infection surveillance utilize culture-based diagnostics, which can only identify microorganisms with the physical characteristics of sedimentation and easy cultivation. Thus, the surveillance of hospital infections with this method is insufficient for a broad coverage of airborne pathogens and does not reflect the real hospital environment. Recent technological advances have driven the development of large-scale tests performed with a single collection that complement and, in some cases, replace the traditional methods^13^,^14^,^15^ of detection. In particular, the development of microbiome sequence analysis not only produces higher sensitivities and shorter turnaround times than the traditional methods but may also identify suspended pathogens that are difficult to culture, such as fungi^16^,^17^,^18^.

In this study, we utilized a non-stop high-volume sampler for air collection, with a total collection volume equivalent to the human daily respiratory volume. Next, DNA from the samples was extracted for sequence analysis. After the monitoring and surveillance of air quality, the diversity of airborne fungi was revealed for different departments of the hospital.

## Results

### Few airborne bacteria in the hospital environment can be identified by culture-based technology

**Figure 1:**
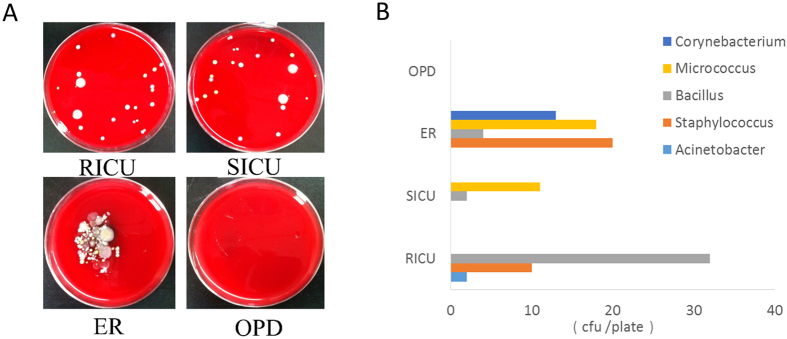
The culture-based study revealed few airborne bacteria in the four departments of the hospital. (**A**) Bacterial growth after cultivation by blood agar. (**B**) Identified bacteria in the different departments.

A positive result in culture-based technology is considered the gold standard for microorganism identification. Airborne microorganisms collected using high-volume samplers and filters from different departments were cultured using standard procedures. Five species of bacteria were grown on blood agar and were identified by observation under a microscope as shown in Fig. 1A. Common bacteria in the environment, including *Micrococcus, Bacillus, Staphylococcus* and *Corynebacterium*, were found in the Emergency Room (ER), three of them were found in the Respiratory Intensive Care Unit (RICU), two of them were found in the Surgical Intensive Care Unit (SICU), and *Staphylococcus* none of them were found in the RICU. The colony-forming units (CFU) of each bacterial species were calculated and are shown in Fig. 1B. The results indicated that the air environment in the Outpatient Department (OPD) was relatively clean. Due to the difficulty of cultivation, and with no pure culture technology available, only bacteria were grown through this traditional culture-based method.

### Airborne microorganisms from different departments can be identified by meta-sequencing-based microbiome analysis in the hospital

**Figure 2:**
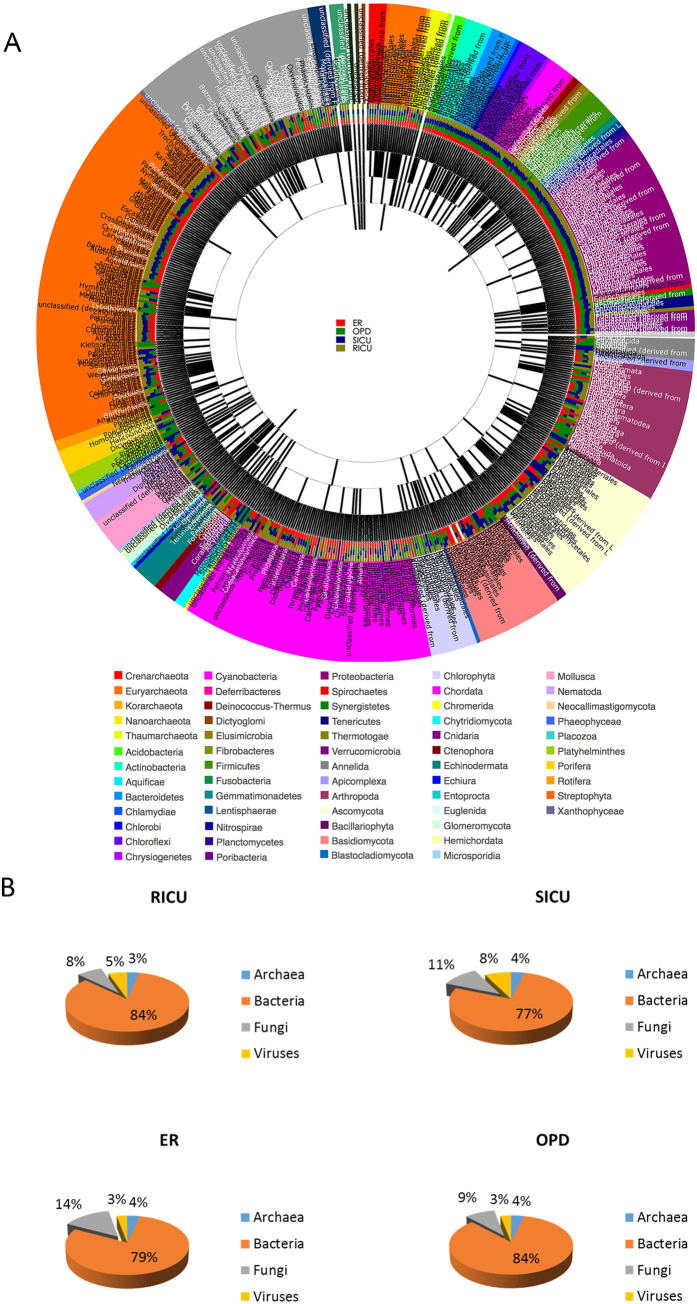
Sequencing analysis revealed numerous microorganisms. (**A**) Maximum Likelihood phylogenetic trees of all 4 conditions. The phylogenetic tree were inferred using the Maximum Likelihood method based on the result of MG-RAST. Different colors of outer circles strand for different families. Inner circle is a stacked bar plot for the four conditions. For the phylogenetic trees, each node represents a taxonomic entity. (**B**) Gene analysis shows the different percentages of fungi in the different departments of the hospital.

Airborne DNA extracted from the four filters was prepared for meta-genomic sequencing. The identified microorganisms were analyzed and divided based on their phylogenetic position (Fig. 2A). This classification of microbe revealed 163 species of fungi (8%), 74 species of Archaea (3%), 1,826 species of bacteria (84%) and 117 species of virus (5%) in the RICU; 314 species of fungi (11%), 106 species of Archaea (4%), 2,170 species of bacteria (77%) and 211 species of virus (8%) in the SICU; 287 species of fungi (14%), 72 species of Archaea (4%), 1,597 species of bacteria (79%) and 60 species of virus (3%) in the ER; and 235 species of fungi (9%), 98 species of Archaea (4%), 2,261 species of bacteria (84%) and 81 species of virus (3%) in the OPD (Fig. 2B). These percentages were calculated by excluding the numbers of other unclassified species. Meta-genomic sequencing identified much higher numbers of species in these departments in comparison to the widely accepted Petri dish culture method.

### A high diversity of fungi is revealed by heatmap analysis

**Figure 3:**
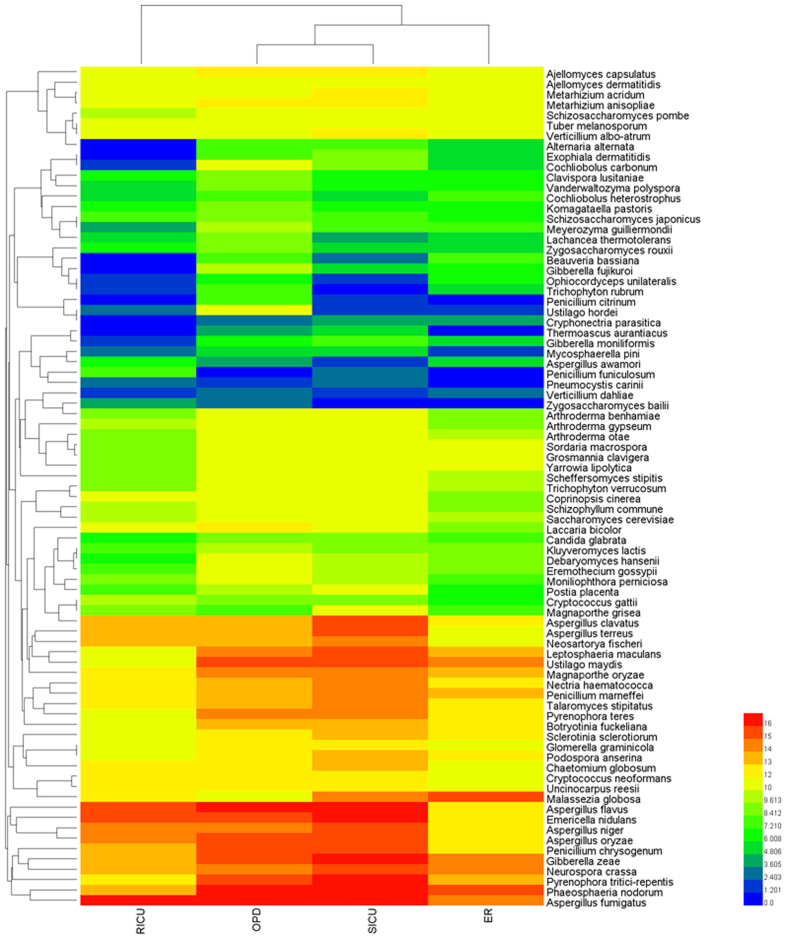
Airborne fungi were revealed by heatmap analysis.

**Figure 4:**
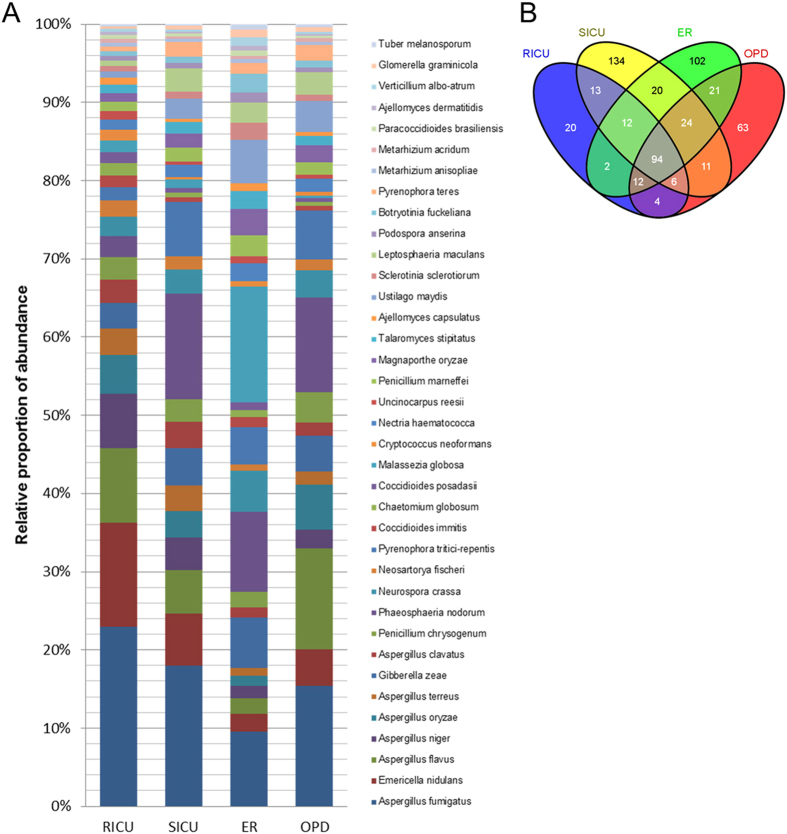
Diversity of the fungal distribution by relative abundance and species intersection relationship. (**A**) For the meta-genomic recovered fungal species, the top 100 abundance species for the heatmap are shown. (**B**) The fungi species intersection of each group in the four different departments.

Using a formula for the plot value equal to log2 (abundance/100), the hit frequency was calculated, and the result was between 10 and −5. The red color represented 10, and the blue color represented −5. The change in color from red to blue represented the frequency of the fungal species in the database shown in Fig. 3. The relative abundance of the identified fungi was calculated, and the proportion is shown in Fig. 4A. At the same time, a cross comparison of these four departments indicated that the components of the identified fungi were dissimilar. The constitution of the fungi species detected in the different departments is shown in Fig. 4B.

### The prevalence of *Aspergillus* differs between departments

**Figure 5:**
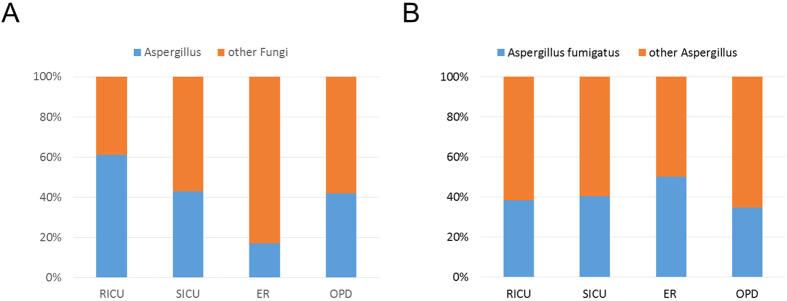
Relative abundance analysis showed the different ratios of *Aspergillus fumigatus* among the fungi in the different departments. (**A**) The different ratios of *Aspergillus* among the fungi in each department were calculated by relative abundance analysis. (**B**) The different ratios of *Aspergillus fumigatus* among *Aspergillus* species in each department were calculated by relative abundance analysis.

The prevalence of *Aspergillus* and the proportion of *Aspergillus* among fungi were calculated, as shown in Fig. 5A. The results revealed that the prevalence of *Aspergillus* among fungi in the RICU reached 61%, which represented the highest level observed among all departments. The second and third highest ratios of *Aspergillus* were 47% and 43%, respectively, in the SICU and OPD. In the ER, the prevalence of *Aspergillus* was 17%. At the same time, the ratio of *Aspergillus fumigatus* among all *Aspergillus* species was calculated. Although the ratio of *Aspergillus* among the total fungi in each department fluctuated in the range of 17–61%, the ratios of *Aspergillus fumigatus* among *Aspergillus* species remained at a relatively fixed level, around approximately 34% to 50% in the investigated departments. The ratio in each department is shown in Fig. 5B. The top 10 fungi identified from the four departments are shown in Supplemental Table 4 at the species level, showing that the top 10 fungi accounted for highest proportion of all identified fungi. The relative abundance of the top 10 fungi is shown in Figure S2, revealing that the ratios of *Aspergillus fumigatus* among all fungi ranked first among all of the identified fungi in the four departments.

## Discussion

The practical importance and frequency of airborne hospital-associated infections remain controversial, although the rate of this type of infection has increased sharply in recent years^19^. Although scientists have worked hard to improve the methods used to monitor infection transmission routes, the surveillance and management of airborne pathogens remain difficult. Special air-sampling devices have been used to collect air for analysis since the 19^th^ century, although it has often been impossible to identify the isolated pathogens accurately because of methodological limitation^20^,^21^. However, new methods, particularly genomic sequencing analysis, have recently emerged to identify and count these pathogens from the air^22^,^23^. These methods can be used to predict the risk of unexpected outbreaks of airborne diseases by identifying differences in the pathogen populations that render control ineffective. In contrast to the small number of pathogens identified using conventional culture-based methods and bypassing the need for isolation and laboratory cultivation of individual strains, sequence analysis can provide a complete picture of the distribution of airborne microorganisms in the hospital environment. The merits of genomic sequencing analysis have not only been demonstrated for the separation of virus species but also for the identification of fungi. The presence of these organisms in the airborne environment exceeded our expectations, especially for fungi. To reflect real respiratory activity, the air sample was set up at an average flow rate of 4 L/min for 24 hours to imitate human breath in this study. We hypothesized that the quantity of microorganisms in the filter was the same as that of human inhalation in one day.

Fungi^24^,^25^ pose three major adverse effects on human health: inflammatory, allergic and toxin effects. The first two types of effect are most commonly exerted through airborne means to human beings^26^. The biggest challenge related to the control of these diseases is that patients are constantly exposed to high concentrations of fungal spores in hospital wards. Therefore, assessing the distribution of airborne fungi in hospitals is particularly important, especially for air condition surveillance.

*Aspergillus* spp. are significant fungal pathogens associated with airway disease^26^. The severity of *Aspergillus* infection depends on the host immune status and can range from a hypersensitivity reaction to fatal invasive pulmonary disease^27^. The spectrum of pulmonary disease associated with *Aspergillus spp.* involves a complex interplay between the respiratory epithelium and the host response in the presence of inhaled spores^25^. In this study, *Aspergillus fumigatus* and other species of *Aspergillus* were identified, including *Aspergillus niger, Aspergillus flavus* and others. At the same time, we found that the relative abundance of *Aspergillus fumigatus* was very high in each department, especially the RICU. This high concentration of *Aspergillus fumigatus* in the RICU may be particularly relevant for patients with previous or permanent broncho-hyperreactivity or with an artificial airway, due to the increased possibility of invasive fungal infection, especially in patients with an opened airway^26^. Consequently, a high frequency of *Aspergillus fumigatus* in the air should be considered unsafe for the treatment of basic diseases. Due to the specificity and distinctiveness of hospital environments, it is important to minimize the amount of air pathogens to which patients are exposed to provide good air quality and to minimize the danger of acquired infection in hospital wards^26^. The study had two main limitations: (1) the study was conducted at a single hospital and (2) we did not establish the relationship between airborne fungi and hospital-acquired invasive fungal infection. In future studies, we would like to focus on the application of this new sequencing method. Furthermore, through long-term observation, we hope that additional questions may be answered.

Surveillance is the core method used to control and manage infections in special units of hospitals^28^. In this study, we utilized a new detection system for monitoring and surveillance of the hospital environment, and our results provide information on the current situation of airborne pathogens in hospitals and about the real microorganisms that patients inhale. Our results show that potentially harmful airborne pathogens are widespread in the hospital, which challenges the results of conventional hospital infection surveillance systems. Addressing the fungal spores suspended in air should be paramount in the control of hospital infections, and increased attention should be focused on these public health issues, including infectious diseases and environmental hazards that can arise^29^. Therefore, fungi diversity has been considered the prominent index to evaluate the indoor air quality (IAQ) by the hospital control manager^26^. Recognition of the limitations of hospital control-surveillance systems may lead to further assessment of the infection prevention programs in different departments of general hospitals, with the aim of providing reliable results to policymakers and health professionals for the development of improved hospital control-surveillance systems^30^.

## Materials and Methods

### Study design

In this study, which was performed in January 2014, all of the samples were collected at Beijing Hospital, a general hospital with more than 500 outpatients per day. Air samplers were set up in 4 departments of the hospital, the Outpatient Department (OPD), Emergency Room (ER), Surgical Intensive Care Unit (SICU) and Respiratory Intensive Care Unit (RICU). The RICU and SICU often are enrolled critical patients and most of the patients were established artificial airway. The Emergency room were enrolled the critical patients who needed emergency treatment. Unfortunately, some of patients must receive medical treatment in ER, due to lack of empty beds in ward. Patients in outpatients department were at high crowd of liquidity. Therefore, patients from the four departments were represented the different danger facing to the airborne fungi threaten.

### Air sample collection

Air sampling pumps (SKC, PA, USA) were set up in the departments mentioned above, and they were connected to a 47-mm, 0.2-μm PTFE membrane filter holder (PALL, NY, USA) by a TYGON tube (Saint-Gobain Corporation, USA). Each air sampling pump contained two filters. The filters used to collect air samples were sterilized by autoclaving following the user’s guide. Each sterilized filter was packaged in sterilized aluminum foil and was stored in a sealed bag until ready for loading into the filter. The filter holder and all of the tools used for changing new filters were cleaned with 75% ethanol or autoclaved each time to avoid contamination. The filter punches were stored at −20 °C. Other samples were stored at −80 °C until downstream analyses were performed. The air sample was drawn at an average flow rate of 4 L/min for 24 hours (4:00 PM to 4:00 PM the next day). The average air flow rate of the human breath is 8 L/min, as reported previously, although half of the air is exsufflated; thus, we used 4 L/min as a cut off for air collection to imitate the real breath situation of a patient in one day^17^.

### Cultivation of the membrane filters

The collected filters were washed with 1 mL of 0.9% NaCl solution and were transferred to cultured plates. The cultured plates were prepared and placed in an incubator in an atmosphere of 5% CO_2_ at 35 °C for 48 hours. Observation of the results was performed every 24 hours. After 48 hours of cultivation, the plates containing between 30 and 300 colonies were used.

### DNA extraction and whole-genome amplification

We applied an optimized protocol for extracting limited quantities of airborne microbial genomic DNA for direct metagenomic sequencing^31^. The membrane filters were carefully removed from the filter holders and were cut into small pieces. The cutting membranes were placed into 15-mL sterilized centrifuge tubes to which 1 mL of ddH_2_O was added. After vortexing gently for 30 minutes, the upper liquid containing DNA was transferred into a sterilized Eppendorf tube. DNA extraction was performed according to the standard protocol of the MO-BIO PowerSoil Mini DNA Isolation Kit (Carlsbad, CA, USA). Next, the DNA samples were eluted into 10 μL TE buffer (Thermofisher, CA, USA) and then were subjected to whole-genome amplification using the REPLI-g^®^ Single Cell Kit (Qiagen, Hilden, Germany) to generate enough DNA for sequence library construction. Blank control samples were collected by placing a sterilized filter inside of the sampler without operation for 23 hours, followed by treatment as described above. The whole-genome amplification of samples from the four departments and blank control samples was performed using a StepOne Real-time PCR machine (Life Technologies, CA, USA). All of the DNA samples were stored at −80 °C for further usage.

Accurate DNA quantification was obtained using a Qubit^®^ 3.0 Fluorometer (Thermofisher, CA, USA). The DNA libraries were prepared according to the Enzymatics user manual (Enzymatics, MA, USA). The size distribution of the libraries was evaluated using a 2100 Bioanalyzer (Agilent, CA, USA), and DNA quantification was obtained using 7500 Real Time PCR (Thermfisher, CA, USA). Paired-end sequencing (2 × 90 bp) with an insert size of ~500 base pairs was performed on a HiSeq 2500 machine (Illumina, CA, USA).

### Bioinformatics and statistical analysis of metagenomes

The fastq files generated by Illumina sequencing were qualitatively evaluated using FASTQC (v.0.11.2, http://www.bioinformatics.babraham.ac.uk/projects/fastqc/). Adaptor contamination and low-quality reads were discarded from the raw data with Trimmomatic^32^. Sequence annotation was conducted via Metagenome Rapid Annotation using the Subsystem Technology (MG-RAST) webserver (http://metagenomics.nmpdr.org/)^33^. Compared with M5NR, the alpha diversity of the four different departments was calculated using a maximum e-value of 1e-5, a minimum identity of 60%, and a minimum alignment length of 15 (measured in amino acids (aa) for protein and bp for RNA). Taxonomic annotation was performed using the GenBank database, (http://www.ncbi.nlm.nih.gov/) and functional annotation was completed using the SEED database^34^. The statistical analyses were performed with R version 3.0.3 (https://www.r-project.org/). The visualization of phylogenetic tree was draw by uploading the data to iTOL^35^. The heatmap and venn diagrams were also drawn with R package (https://www.r-project.org/). The metagenomic sequencing data can directly access from MG-RAST website under accession number 9266 and 8353.

### Real-time quantitative PCR

The samples were analyzed using the iQ5 Multicolor Real-Time PCR Detection System (Bio-Rad Laboratories, Hercules, CA, USA) to identify fungi species. Real-time PCR was performed using SYBR^®^ Premix Ex TaqTM II (Takara, Dalian, China) according to the manufacturer’s protocol. The two-step PCR protocol included one cycle at 95 °C for 30 seconds, followed by 40 cycles at 95 °C for 5 seconds and 60 °C for 30 seconds. The PCR reaction required 9.9 μL of distilled water, 12.5 μL of 2 × SYBR^®^ Premix Ex TaqTMII, 0.8 μL of each for primers, and 1 μL of DNA template in a total volume of 25 μL. All of the reactions were run in triplicate. The Ct values for the relative quantification of gene expression were used to determine the fungi read expression levels. The sequences of the PCR primers are shown in Supplemental Table 2, and the amplification chart for real-time PCR is shown in Supplemental Figure 1. To verify the candidate fungi species, the total genomic DNA of the 4 meta-genome samples was prepared as the template for real-time PCR to determine the abundance of fungi. The CT value represented the increased quantization of the product, as shown in Supplemental Table 3.

## Additional Information

**How to cite this article:** Tong, X. *et al*. High diversity of airborne fungi in the hospital environment as revealed by meta-sequencing-based microbiome analysis. *Sci. Rep.* **7**, 39606; doi: 10.1038/srep39606 (2017).

**Publisher's note:** Springer Nature remains neutral with regard to jurisdictional claims in published maps and institutional affiliations.

## Supplementary Material

Supplementary Information
