# Supplementary material for: High diversity of airborne fungi in the hospital environment as revealed by meta-sequencing-based microbiome analysis
Source: Sci Rep. 2017 Jan 3;7:39606. doi: 10.1038/srep39606 (PMC5206710; doi:10.1038/srep39606)
Supplement: Supplementary Information [file srep39606-s1.pdf]

High diversity of air borne fungi in hospital environment revealed by meta-sequencing based microbiome analysis

Xunliang Tong [a¶], Hongtao Xu [b¶], Lihui Zou [c], Meng Cai [d], Xuefeng Xu [e], Zuotao Zhao[f], Fei Xiao [c] and Yanming Li [d, g]

[a] Department of Geriatrics, Beijing Hospital, Beijing, the People's Republic of China; [b] Department of Laboratory Medicine, Beijing Hospital, Beijing, the People's Republic of China; [c] Key Laboratory of Geriatrics, Beijing Institute of Geriatrics, Beijing Hospital, Beijing, the People's Republic of China; [d] Department of Hospital Infection Control and Management, Beijing Hospital, Beijing, People's Republic of China; [e] National Clinical Research Centre for Respiratory Medicine, Beijing Hospital, Beijing, the People's Republic of China; [f] Department of Dermatology, First Hospital, Peking University, Beijing, the People's Republic of China; [g] Department of Respiratory and Critical Care Medicine, Beijing Hospital, Beijing, the People's Republic of China;

[¶]Xunliang Tong and Hongtao Xu contributed equally to this work.

#Address correspondence to Dr. Yanming Li, lymyl@263.net.

Running Head: Diversity of air borne fungi in hospital environment

Supplement Materials

Figure. S1 The amplification chart of real-time PCR

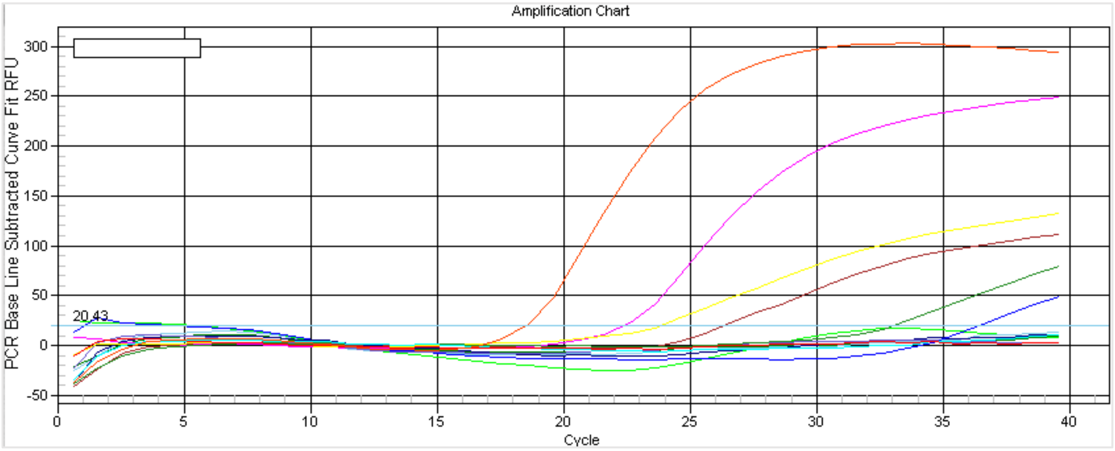

Figure. S2 Relative abundance of top 10 identified fungi were different in the four departments of hospital

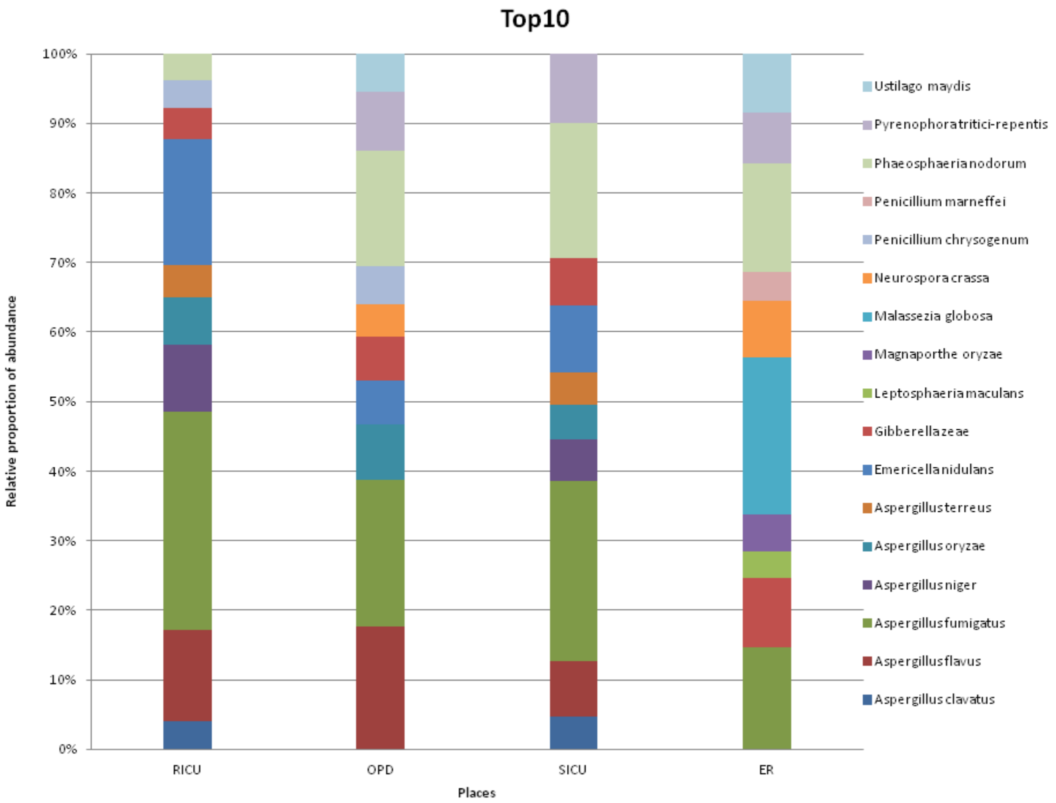

Table. S1. Clean bases of sequence data were generated from the different departments

	Raw_reads	Raw_bases	Clean_reads	Clean_bases	Ratio
SICU	174,474,182	174474182101	173,728,752	17,546,603,952	99.57%
RICU	128,418,162	12,970,234,362	128,133,412	12,941,474,612	99.78%
OPD	45,966,580	4,639,099,292	45,829,914	4,625,305,072	99.70%
ER	21,335,980	2,153,106,418	21,229,302	2,142,349,120	99.50%

Table. S2. Primers information of real time PCR

	Forward Primer	Reverse Primer
Cryptococcus	TGATGAGCGTGTATTCCGGC	CATGCGAATGAGAGTTTCTAG
Emericella	TCACCGTCGCCACTCTCGTC	GACAACCTTCTTGATCTGTC
Malassezia	CATCTCGACTGTTCTTGCGC	GCTCTCGAGAGCCGTGGGCG
Chrysogenum	CGCGGCGCTGCTTCCATTCG	CGAGAGAATCGGTACGGAGA
Marneffei	ACCAAGCCCGCTATCCGCCG	TCGAGGAAGGTCTTGAGGAC
Aspergillus fumigatus	ATTCGCTGGTGGTCACCCAC	ATACGACCATAGGGTGTGGA

Table. S3. Values of CT were shown that the quantization of DNA product was significantly increased in samples

	Ct Values
Cryptococcus Neoformans	23.88
Emericella Nidulans	30.93
Peniaillium Marneffei	18.49
Aspergillus Fumigatus	15.33

Table. S4. Abundance of top10 identified fungus was different in the four departments of hospital

Species	RICU Abundance	SICU Abundance	ER Abundance	OPD Abundance
Aspergillus fumigatus	92,935	266,881	31,372	142,175
Emericella nidulans	53,768	99,495	7,429	42,802
Aspergillus flavus	38,670	82,325	6,624	118,661
Aspergillus niger	28,113	61,475	5,282	21,883
Aspergillus oryzae	20,070	50,860	4,124	53,740
Aspergillus terreus	13,806	48,846	3,262	15,052
Gibberella zeae	13,243	70,946	21,215	42,217
Aspergillus clavatus	11,962	49,112	4,375	15,388

Penicillium chrysogenum	11,678	43,764	6,555	36,205
Phaeosphaeria nodorum	11,187	200,185	33,489	111,441
Other fungus	123,700	551,860	224,197	374,441
